# Continental-Mediterranean and rural-urban differences in cardiovascular risk factors in Croatian population

**DOI:** 10.3325/cmj.2011.52.566

**Published:** 2011-08

**Authors:** Biserka Bergman Marković, Davorka Vrdoljak, Ksenija Kranjčević, Jasna Vučak, Josipa Kern, Ivan Bielen, Dragica Ivezić Lalić, Milica Katić, Željko Reiner

**Affiliations:** 1 Department of Family Medicine, Zagreb University School of Medicine, Zagreb, Croatia; 2 Department of Family Medicine, University of Split School of Medicine, Split, Croatia; 3 General Practice Office, Zagreb-zapad Health Care Center, Zagreb, Croatia; 4 General Practice Office, Sukošan, Croatia; 5 Department of Medical Statistics, Epidemiology and Informatics, University of Zagreb School of Medicine, Zagreb, Croatia; 6 Department of Neurology, Holy Ghost, University Hospital, Zagreb; 7 General Practice Office, Novska, Croatia; 8 Department of Internal Medicine, University of Zagreb Hospital Center, University of Zagreb School of Medicine, Zagreb, Croatia

## Abstract

**Aim:**

To compare the distribution of cardiovascular disease (CVD) factors between continental and Mediterranean areas and urban and rural areas of Croatia, as well as to investigate the differences in achieving treatment goals by the general practitioners (GP) in different settings.

**Methods:**

A multicenter prospective study was performed on 2467 participants of both sexes ≥40 years old, who visited for any reason 59 general practices covering the whole area of Croatia (May-July 2008). The study was a part of the Cardiovascular Risk and Intervention Study in Croatia-family medicine (CRISIC-fm) study. Patients were interviewed using a 140-item questionnaire on socio-demographics and CVD risk factors. We measured body mass index (BMI) and waist circumference and determined biochemical variables including blood pressure, total, high-density lipoprotein-, and low-density lipoprotein-cholesterol, triglycerides, glycemia, and uric acid.

**Results:**

Participants from continental rural areas had significantly higher systolic and diastolic blood pressure (*P* < 0.001), obesity (*P* = 0.001), increased waist circumference (*P* < 0.001), and more intense physical activity (*P* = 0.020). Participants from coastal rural areas had higher HDL-cholesterol, participants from continental rural and coastal urban areas had higher LDL-cholesterol, and participants from rural continental had significantly higher BMI and waist circumference.

**Conclusion:**

Prevalence of CVD risk factors in Croatian population is high. Greater burden of risk factors in continental region and rural areas may be partly explained by lifestyle differences.

Cardiovascular disease (CVD) is the major cause of death in the developed world but also an increasingly important cause in developing and underdeveloped countries that are adopting the Western way of life (1). In Europe, there was an increase in CVD mortality from north to south, but recently an increase from west to east has been observed. Still, the lowest mortality rates are mainly found in the Mediterranean countries (2). Although Croatia is geographically a Mediterranean country, its CVD mortality rate is significantly higher than in other Mediterranean countries, and in fact it is closer to Central and Eastern Europe (Croatian paradox) (2,3). CVD is a leading cause of death in Croatia and contributes to nearly 50% of total mortality cases (4).

Only a few epidemiological studies on CVD risk factors in general population of Croatia have been performed over the last 50 years. A study performed in 1995-1997 did not investigate anthropometric data on central obesity (5), while Croatian Adult Health Survey performed in 2003 did not include a laboratory analysis of important CVD risk factors (6).

Although there was one prevalence study on hypertension in Croatian general population (7), two studies on risk factors on hospitalized patients with CVD (8,9), and one small survey on high risk persons as a part of a large multinational study (10), there has been no comprehensive study of the CVD risk factors and total CVD risk in a representative sample of the adult general population in Croatia. There was also only one study comparing CVD risk factors in different regions of Croatia but it was performed on hospitalized patients with CVD eight years ago (11).

The aim of our study was to investigate the distribution and possible differences among CVD risk factors (elevated blood pressure, dyslipidemia, hyperglycemia, hyperuricemia, smoking, overweight, obesity and central obesity, physical activity, alcohol consumption, total CVD risk calculated by SCORE and by Framingham) between different parts of Croatia (continental-inland and Mediterranean-coastal) and different settlement sizes (urban/rural), as well as to determine whether the target values of CVD risk factors (blood pressure, serum lipids, blood glucose) according to the guidelines (12) are achieved in the primary and secondary CVD prevention.

## Methods

### Study design

This was a cross-sectional arm of a two-phase, multicenter, prospective, cohort, cluster randomized, intervention study of CVD risk factors conducted in the general practitioners' (GP) practices covering all parts of Croatia. The study was named Cardiovascular Risk and Intervention Study In Croatia-family medicine (CRISIC-fm) and it was registered as a clinical trial (International Standard Randomized Controlled Trial Number Register – ISRCTN31857696). In the first phase, representative sample of 59 GP’s offices was created and in the second phase, each GP included 55 consecutive participants ≥40 years old who visited his/her practice for any reason and signed informed consent. The study was approved by the Ethics Committee of Zagreb University School of Medicine.

### Participants

The study was conducted in 59 GP's practices covering the whole area of Croatia between May and July 2008. It enrolled 2467 participants of both sexes. The exclusion criteria included communication disability (dysphasia, aphasia), severe dementia, and non-cardiac disease with estimated life expectancy of less than six months.

### Sampling

In the first phase, the 4-stage stratified representative sample of general practices in different parts of Croatia was created based upon counties (Croatia is divided into 21 counties), region (coastal or continental), settlement size (up to 3999 inhabitants, 4000 to 9999, 10 000 to 29 999, 30 000 to 89 999, and 90 000 inhabitants and more), and the number of insured subjects in a GP’s care in 2007 based on a list of all GP’s/family medicine practices having a contract with the Croatian Institute for Health Insurance (national compulsory health insurance system covering 97% of the population) in 2007.

Coastal region was defined as the area 30 km from the sea if there was no natural obstacle (mountains). Settlements with fewer than 4000 inhabitants were defined as rural, while those with more were defined as urban. Within each stratum, GP's offices were randomized and selected by the random number generator.

### Study size

The initial participants’ sample size for each general practice was based upon power analysis – criterion of power of at least 80% and reaching 95% confidence interval to be representative for all GPs in Croatia. When calculating the power, the prevalence of CVD risk factors in Croatia, according to available data, was taken into account, as well as a methodological factor of data dispersion (this level was set arbitrarily and according to the previous pilot studies conducted in family practice in Croatia to 20%). The minimum number of 55 participants was determined for each GP to be sufficient for achieving the study aims and the credibility of conclusion for the entire population aged ≥40 years in GP’s care.

### Measurements

A 140-item CRISIC-fm questionnaire was created. The questionnaire collected sociodemographic information and data on CVD risk factors, diet, physical activity, and alcohol consumption.

The analyzed parameters were body height and weight (the mean of two measurements on standardized anthropometric scales), waist and hip circumference (by plastic coated, non-elastic tape), and arterial blood pressure (the mean of two measurements performed by mercury sphygmomanometer).

Blood samples were taken from fasting participants, and the following was measured: total cholesterol, high density lipoproteins (HDL) and low density lipoproteins (LDL) fraction, triglycerides, blood glucose, and uric acid concentrations. Diagnostic criteria and target goals for blood pressure and lypidemia in primary prevention and in patients with already established cardiovascular disease, ie, secondary prevention were based on European Joint Prevention Guidelines (12) and European Society of Cardiology-European Society of Hypertension guidelines (13). Target blood pressure in primary prevention was <140/90 mm Hg, cholesterolemia <5.0 mmol/L, LDL<3.0 mmol/L, and in secondary prevention <130/80 mm Hg, cholesterolemia <4.0 mmol/L, LDL<2.5 mmol/L.

Blood glucose levels and pre-diabetes were defined according to the American Diabetes Association guidelines (14), the metabolic syndrome according to the International Diabetes Federation guidelines (15), overweight, obesity, waist circumference, and waist-to-hip ratio according to WHO (16). Central obesity was defined as waist circumference >88 cm for women and >102 cm for men. Physical activity was defined according to American College of Sports Medicine/American Heart Association guidelines (17).

Ten-year risk of fatal CVD was calculated using the Systematic Coronary Risk Evaluation SCORE chart for high-risk countries (18) and the Framingham Table for stroke (19).

Moderate drinking was defined as ≤20 g ethanol per day for men and ≤10 g for women. “Mediterranean diet” meant a daily intake of fruits and vegetables, brown bread and whole grains, using olive oil as the main source of fat, consumption of fish and moderate wine drinking with meals (20).

### Distortions

Standard error of measurement was reduced by using the identical standardized measuring instruments at all locations and by repeated measurements (2 × ). Numerical data verification and logical control of systematic errors were carried out.

### Statistical analysis

Descriptive statistical methods were used to describe the basic characteristics of the sample of participants. Frequencies of basic characteristics are presented by contingency tables. To test the relationships between two categorical variables (continental/coastal) and settlement size (rural/urban), we used χ^2^ test. The effect of the interaction between regions and the settlement size on the dependent variables (CVD risk factors) was tested for each one by two-way ANOVA. All values were interpreted at the significance level of 95% (*P* < 0.05). All statistical methods were performed using SPSS for Windows, version 11.5 (SPSS Inc., Chicago, IL, USA).

## Results

### Patient demographics

**Figure 1:**
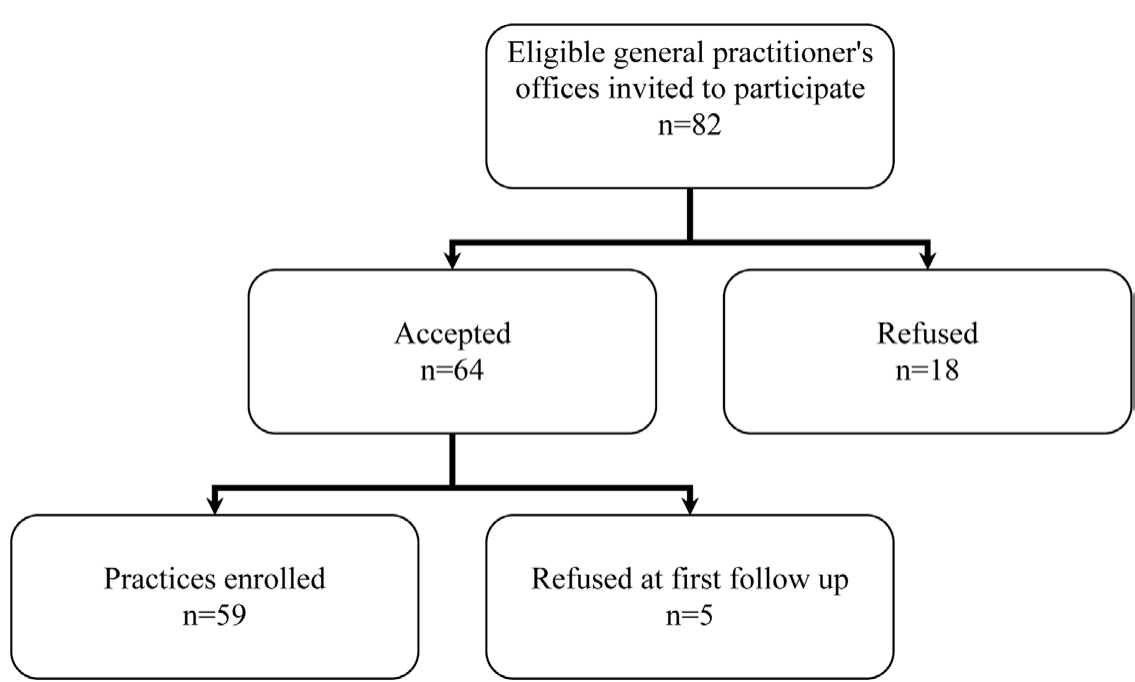
The enrolment of general practitioners' offices into the study.

**Figure 2:**
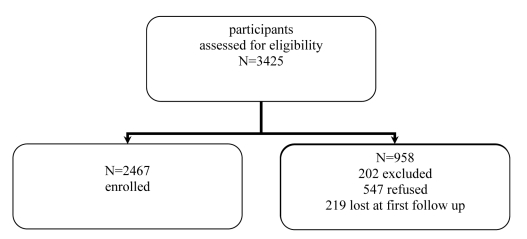
The enrolment of patients into the Cardiovascular Risk and Intervention Study in Croatia-family.

Fifty nine general practices were included (response rate 70%) with 2467 patients (response rate 71%) (Figures 1 and 2). There were more women than men (Table 1). Most of the participants (37%) belonged to the age group 40-54 years, had high-school education, and were mostly retired, workers, farmers, or housewives. Monthly household income in continental rural areas was well below average when compared with other regions (Table 1).

**Table 1:** Study sample description according to urban/rural residence after stratification for region (continental/coastal) in 2467 Croatian adults who visited general practitioner's office from May to June 2008

	No. (%) of participants from the area				
	continental		coastal		P*
	urban (n = 1243)	rural (n = 465)	urban (n = 581)	rural (n = 177)	
Sex:					
men	444 (37.0)	191 (41.2)	229 (39.5)	58 (32.8)	0.172
women	757 (63.0)	273 (58.8)	350 (60.5)	118 (67.2)	
no data for 47 (1.9) participants					
Age (years):					
≤54	457 (37.2)	172 (37.0)	222 (38.4)	59 (33.4)	0.793
55-64	398 (32.4)	150 (32.3)	195 (33.8)	58 (32.5)	
≥65	374 (30.4)	143 (30.7)	161 (27.8)	60 (34.1)	
no data for 18 (0.7) participants					
Partnership status:					
living alone	277 (23.3)	109 (23.8)	137 (23.9)	38 (21.5)	0.935
living with partner	910 (76.7)	350 (76.2)	437 (76.1)	138 (78.5)	
no data for 72 (2.9) participants					
Education:					
unfinished primary school	51 (4.3)	81 (17.6)	39 (6.7)	30 (17.2)	<0.001
finished primary school	220 (18.4)	160 (34.4)	98 (16.9)	40 (22.9)	
high school	627 (52.3)	181 (39.1)	311 (53.8)	84 (47.6)	
polytechnic	135 (11.2)	23 (4.9)	76 (13.1)	14 (7.9)	
university	165 (13.8)	18 (4.0)	55 (9.5)	8 (4.4)	
no data for 49 (2.8) participants					
Profession:					
housewife	117 (9.8)	109 (23.5)	79 (13.7)	38 (21.5)	<0.001
worker or farmer	249 (20.8)	99 (21.3)	139 (24.1)	41 (23.1)	
craftsman	57 (4.8)	15 (3.1)	40 (6.9)	4 (2.4)	
manager	72 (6.1)	15 (3.2)	40 (6.8)	5 (2.9)	
retired	582 (48.7)	182 (39.3)	241 (41.7)	76 (43.0)	
unemployed	59 (5.0)	34 (7.4)	22 (3.8)	9 (4.9)	
other	59 (4.9)	10 (2.1)	17 (3.0)	4 (2.3)	
no data for 49 (2.0) participants					
Household monthly net income (€ per month):^†^					
considerably below the average (up to 414)	215 (18.4)	135 (29.5)	97 (16.9)	41 (23.6)	<0.001
somewhat below the average (up to 550)	287 (24.5)	122 (26.8)	154 (26.9)	48 (27.5)	
about the average (up to 830)	288 (24.6)	98 (21.4)	113 (19.6)	42 (23.7)	
somewhat above the average (up to 1170)	212 (18.1)	60 (13.1)	120 (20.9)	24 (13.9)	
considerably above the average (up to 1380)	169 (14.4)	42 (9.3)	90 (15.7)	20 (11.4)	
no data for 89 (3.6) participants					

*χ^2^ test.

†According to Republic of Croatia Central Bureau of Statistics during November 2009 the average Croatian net income was €750.

### CVD factors

There were 1015 (42%) overweight participants, 875 (36%) were obese, and 1292 (54%) had central obesity. In continental rural areas, there was a significantly higher prevalence of increased systolic and diastolic blood pressure in participants without CVD (*P* < 0.001), as well as in those with CVD or diabetes (*P* < 0.001), and higher blood glucose >6.0 mmol/L in those with CVD or diabetes (*P* = 0.026). This population also had more frequent metabolic syndrome (*P* < 0.001), obesity (*P* = 0.001), and increased waist circumference (*P* < 0.001) than population in the coastal region.

There was no difference in total CVD risk calculated by SCORE and Framingham score between the participants living in continental/coastal or urban/rural areas. However, the prevalence of CVD factors was higher among patients with CVD or diabetes than among patients without CVD or diabetes (Table 2).

**Table 2:** Cardiovascular risk factors according to urban/rural residence after stratification for region (continental/coastal) in 2467 Croatian adults who visited general practitioner's office from May to June 2008

	No. (%) of participants from the area				
	continental		coastal		
	urban (n = 1243)	rural (n = 465)	urban (n = 581)	rural (n = 177)	
**Participants without cardiovascular disease or diabetes**					
**Systolic blood pressure (mm Hg)>140 or therapy**	**711 (60.0)**	**329 (72.2)**	**236 (42.0)**	**96 (54.7)**	**<0.001**
**Diastolic blood pressure (mm Hg)>90 or therapy**	**682 (57.6)**	**317 (69.5)**	**204 (36.4)**	**81 (46.4)**	**<0.001**
**Total cholesterol (mmol/L)>5.0 or therapy**	**933 (80.7)**	**350 (78.8)**	**432 (79.0)**	**130 (77.9)**	**0.712**
**Low density lipoprotein cholesterol (mmol/L)>3.0 or therapy**	**783 (76.8)**	**311 (79.1)**	**354 (74.9)**	**77 (70.2)**	**0.184**
**Fasting blood glucose (mmol/L)>7.0**	**151 (12.8)**	**64 (14.1)**	**86 (15.3)**	**20 (11.4)**	**0.452**
**High density lipoprotein cholesterol (mmol/L) – low^†^**	**188 (22.1)**	**64 (21.5)**	**71 (17.8)**	**18 (15.0)**	**0,131**
**Triglycerides (mmol/L)>1.7**	**396 (44.0)**	**162 (48.1)**	**179 (42.6)**	**57 (42.5)**	**0.453**
**Participants with cardiovascular disease or diabetes**					
**Systolic blood pressure (mm Hg)>130 or therapy**	**796 (67.2)**	**356 (78.0)**	**300 (53.5)**	**114 (65.1)**	**<0.001**
**Diastolic blood pressure (mm Hg)>80 or therapy**	**788 (66.5)**	**360 (78.9)**	**273 (48.7)**	**117 (67.6)**	**<0.001**
**Total cholesterol (mmol/L)>4.5 or therapy**	**1087 (92.3)**	**411 (90.3)**	**499 (89.7)**	**162 (93.9)**	**0.141**
**Low density lipoprotein cholesterol (mmol/L)>2.5 or therapy**	**870 (84.9)**	**355 (89.7)**	**392 (82.2)**	**93 (82.3)**	**0.016**
**Fasting blood glucose (mmol/L)**	**400 (34.0)**	**165 (36.2)**	**182 (32.5)**	**41 (23.7)**	**0.026**
High density lipoprotein cholesterol (mmol/L) – low^†^	261 (23.6)	85 (22.1)	99 (19.6)	24 (15.9)	0.074
Triglycerides (mmol/L)>1.7	538 (46.6)	220 (49.7)	249 (45.6)	78 (45.3)	0.588
Hyperuricemia^‡^	946 (89.1)	330 (85.1)	443 (89.4)	140 (87.3)	0.146
Prediabetes fasting blood glucose >5.6-6.9 (mmol/L)	473 (46.5)	473 (46.5)	159 (34.6)	39 (25.6)	<0.001
Metabolic syndrome	735 (59.6)	336 (72.3)	322 (56.2)	107 (61.3)	<0.001
Body mass index (kg/m^2^):					
underweight	14 (1.2)	9 (1.9)	11 (1.9)	2 (0.9)	
normal	252 (21.1)	69 (14.9)	126 (21.7)	39 (22.4)	
overweight	509 (42.5)	175 (38.1)	261 (44.9)	70 (40.2)	
obese	421 (35.2)	208 (45.1)	183 (31.5)	64 (36.4)	0.001
Waist circumference (cm) – men ≥102; women ≥88	628 (52.8)	295 (63.5)	279 (48.8)	91 (52.3)	<0.001
Waist-to-hip ratio – men ≥1, women ≥0.85	361 (40.3)	172 (43.3)	178 (42.5)	50 (36.4)	0.422
Estimated total risk:					
SCORE risk >5% risk^§^	604 (72.4)	259 (72.5)	322 (78.1)	100 (80.3)	0.054
Framingham risk>10%^║^	673 (75.9)	182 (74.5)	232 (77.9)	93 (77.3)	0.816

*χ^2^ test.

†Men <1.1 mmol/L, women <1.3 mmol/L.

‡Men >430, women >360 μmol/L.

§According to Conroy et al (18).

Average levels of total and LDL-cholesterol and body mass index (BMI) were higher than target goals, whereas the values of blood pressure were within target values range in the whole sample (Table 3). Regional differences in other CVD risk factors were found: there were more participants with mild and moderate physical activity, excessive drinkers, and non smokers among the continental rural inhabitants. (Table 4).

**Table 3:** Cardiovascular risk factor distribution in 2467 Croatian adults who visited general practitioner's office from May to June 2008, according to region (continental/coastal) and urban/rural area (mean and 95% confidence intervals)

	Continental		Coastal	
	urban	rural	urban	rural
Systolic blood pressure (mm Hg)	130.9 (129.8-132.0)	134.5 (132.5-135.5)	128.8 (127.5-130.3)	134.9 (132.4-137.4)
Diastolic blood pressure (mm Hg)	80.2 (79.6-80.8)	81.8 (81.0-82.6)	79.6 (78.8-80.3)	82.1 (80.7-84.5)
Total cholesterol (mmol/L)	5.8 (5.7-5.9)	5.8 (5.6-5.9)	5.8 (5.7-5.9)	5.8 (5.6-6.0)
High density lipoprotein cholesterol (mmol/L)	1.5 (1.5-1.6)	1.5 (1.5-1.6)	1.5 (1.5-1.6)	1.7 (1.6-1.8)
Low density lipoproteins cholesterol (mmol/L)	3.4 (3.4-3.5)	3.6 (3.5-3.7)	3.5 (3.4-3.6)	3.3 (3.0-3.5)
Triglycerides (mmol/L)	1.8 (1.8-1.9)	1.9 (1.8-2.1)	1.9 (1.8-2.0)	1.7 (1.5-1.9)
Uric acid (μmol/L)	285.0 (278.7-291.4)	292.3 (283.0-301.6)	290.5 (282.2-298.8)	281.6 (266.5-296.8)
Fasting blood glucose (mmol/L)	6.0 (5.4-6.7)	6.9 (6.0-7.8)	5.9 (5.0-6.7)	5.7 (4.2-7.2)
Body mass index (kg/m^2^)	28.7 (28.0-29.4)	30.9 (29.9-31.9)	28.3 (27.4-29.2)	28.6 (26.9-30.2)
Waist-hip-ratio	0.89 (0.88-0.90)	0.91 (0.90-0.93)	0.88 (0.87-0.91)	0.92 (0.89-0.95)

**Table 4:** Habits of 2467 Croatian adults who visited general practitioner’s office from May to June 2008.

	No. (%) of participants from the area:				
	continental		coastal		
	urban (n = 1243)	rural (n = 465)	urban (n = 581)	rural (n = 177)	P*
Physical activity:					
mild	433 (39.0)	139 (32.8)	221 (42.3)	64 (38.1)	0.020
moderate	642 (57.8)	260 (61.3)	286 (54.7)	100 (59.5)	
intensive	36 (3.2)	25 (5.9)	16 (3.1)	4 (2.4)	
Mediterranean diet^†^	13 (1.0)	0 (0.0)	14 (2.4)	6 (3.4)	<0.001
other	1230 (99.0)	465 (100.0)	567 (97.6)	170 (96.6)	
Alcohol:					
<10 g/d	73 (27.4)	14 (12.0)	30 (14.6)	3 (5.4)	<0.001
10-20 g/d	35 (13.2)	9 (7.7)	13 (6.3)	5 (8.9)	
>20 g/d	158 (59.4)	94 (80.3)	162 (79.0)	48 (85.7)	
Cigarette smoking:					
non smoker	578 (48.4)	303 (65.4)	268 (46.5)	97 (54.8)	<0.001
ex smoker	374 (31.3)	100 (21.6)	186 (32.3)	47 (26.6)	
current smoker	243 (20.3)	60 (13.0)	122 (21.2)	33 (18.6)	

*χ^2^ test.

†Mediterranean diet (21).

### Differences in CVD factors according to regions and settlement type

There was a significant influence of life in rural settlements (both continental and coastal) on systolic and diastolic blood pressure (effect of the settlement size, *P* < 0.001; region, *P* < 0.001) and of settlement size on HDL-cholesterol in participants from rural areas (*P* = 0.004). However, this was due to a significant interaction effect between settlement size and region (*P* = 0.015), indicating that participants from rural coastal areas had higher HDL-cholesterol than all the other participants (Table 3). A significant interaction effect was also shown between region and settlement size for LDL-cholesterol: the levels were higher in continental rural and coastal urban settlements (*P* = 0.004) (Table 3). The region (*P* = 0.020) and settlement size (*P* = 0.029) were respectively associated with BMI, so that participants from rural settlements in the continental area had a higher BMI. A significantly higher waist-to-hip ratio (*P* = 0.001) was found in rural than in urban settlements.

## Discussion

Our study demonstrated a high prevalence of CVD risk factors in the Croatian population, with a greater burden of risk factors in continental region and rural areas.

The limitations of the study include the characteristics of the sample, which consisted of only the participants who visited GP’s offices, who do not fully reflect the average Croatian population over 40 years of age. Furthermore, all patients who met the inclusion criteria did not consent to participate in the study. Also, participants without health insurance (the number is almost negligible in Croatia) or those who do not visit GPs were not included.

### Geographical variations in CVD factors

Our study showed significant differences in CVD factors between two different climatic and geographic regions – continental and Mediterranean, as well as the differences between urban and rural areas. The differences were found in systolic and diastolic blood pressure, obesity and waist circumference (more common in rural continental areas), and HDL-cholesterol (higher in the coastal rural areas) and might be explained by different lifestyles, nutrition habits, and poorer accessibility of health care in rural than in urban areas.

We did not find a difference in total cholesterol concentration between Mediterranean and continental parts of Croatia, which does not correspond to data the obtained in the study conducted in seven countries (22). Increased LDL-cholesterol and triglycerides in coastal urban areas and continental rural areas can be explained by recent changes in the nutrition pattern and population migration (23). The data obtained in this survey indicate that residents of coastal area tend to take over the less healthy dietary habits of the continental region and that traditional Mediterranean diet is replaced by “westernized diet” and “junk food.” Significantly higher HDL-cholesterol in the rural coastal area is most probably a consequence of more intense physical activity.

It has already been shown that glucoregulation in diabetic patients with CHD in Croatia is not satisfactory (9). According to this study, it was especially poor in coastal urban areas in participants without CVD, while it was worst in the continental areas in participants with CVD. The reason for this is to a lesser extent GP’s “therapeutic inertia,” and to much greater extent administrative obstacles inherent to the Croatian health care system, which restricts the initiation of insulin therapy by the GPs. Namely, a GP cannot initiate the insulin treatment without prior referral to a diabetologist, and there are not enough of them, particularly in rural areas.

About 40% of participants fulfilled the diagnostic criteria for prediabetes, which is a high percentage. Prediabetes demands GP’s intervention in order to prevent or delay the appearance of “real” diabetes and its complications. Consequently, better care for patients with prediabetes/diabetes should be one of the national health priorities in Croatia.

There were fewer than a third of current smokers in this study, with a greater proportion in the coastal area and urban surroundings. Taking into account the limitation of the study sample, the number of smokers in Croatia is lower than in previous studies (5,6,24). There has been a decrease in the number of smokers in the west but an increase in the east of Europe (25). Public health campaigns and education should aim at smoking prevention and motivate high risk individuals and patients with CVD to stop smoking.

Overweight and obesity are growing public health problems in the world (25,26), and Croatia is not an exception (21,27). Participants in continental rural areas of Croatia were more often obese and more centrally obese. This can be explained by dietary habits in these parts of Croatia. Their obesity was associated with increased blood pressure and elevated LDL-cholesterol, particularly in patients with CVD or diabetes. This is in agreement with the previous results obtained on CVD patients, suggesting that management of body weight should be given the highest priority in patients with coronary heart disease (27).

Although a very small number of participants in this study, even in the coastal region of Croatia, strictly followed the traditional “Mediterranean diet” (according to the established Mediterranean score) (21), dietary patterns differed regionally and depending on urban/rural surroundings. Mediterranean food pattern can be found in 16 Mediterranean pool countries and has been accepted as healthy diet model (28,29). When combined with appropriate physical activity, it has favorable effect on CVD risk reduction (30) and should be promoted in contrast to nutritional “westernization” trends that are associated with an increased CVD risk.

According to this study, physical activity level depended on the region. This is similar to the findings of other studies in Croatia (31) and abroad (32). Participants from rural surroundings more frequently had intense physical activity, which can be explained by their way of life, while participants from urban surroundings more frequently had mild physical activity. Half of the participants reported no physical activity, which indicates that sport and exercise are not sufficiently integrated into the daily life of the Croatian population.

Only a small percentage of participants admitted regular consumption of alcohol, probably because of social and cultural reasons, embarrassment, and stigma. According to the results of this study, but also to the limited data on this issue in Croatia (33), alcohol is consumed more frequently and in larger amounts by men in the continental and rural areas than in coastal and urban areas. On the other hand, moderate consumption of alcohol (predominantly wine) with meals was more frequent in the rural areas of the coastal region because it is part of the traditional lifestyle and food culture. If moderately taken with meals, alcoholic beverages might even reduce CVD risk (34), while excessive drinking clearly increases it (35).

We found no differences between coastal/continental or urban/rural areas in the 10-year risk of fatal CVD events according to SCORE, or 5-year stroke risk according to Framingham. However, it should be mentioned that this sample consisted mostly of participants with proven CVD who are by definition high-risk patients and are not included in the assessment by SCORE.

### Achieving therapeutic targets of CVD risk factors

The prevalence of hypertension in this study was comparable to previous studies (7). Although there were few hypertensive patients among those without CVD, many of them achieved blood pressure therapeutic targets. Poor blood pressure therapeutic control in high-risk patients with CVD is disturbing, but not surprising because similar results were found in previous Croatian studies and large European surveys (7,22). Although considerable financial resources are being spent on the treatment (36,37), the therapeutic control of blood pressure is not satisfactory (9,38). A possible explanation might be that hypertension often requires treatment with two or more drugs, which considerably reduces compliance, especially in the long term, in CVD patients who also have to take other medications (39).

Although earlier epidemiological studies on the elevated blood pressure prevalence in Croatia used a different methodology (no distinction was made between patients with and without CVD, metabolic syndrome, and diabetes), even they showed a steady increase in the prevalence of elevated blood pressure (5,7). One possible explanation might be improved diagnostics of hypertension. Slightly higher prevalence of elevated blood pressure in this survey, compared to European average (38), can be explained by sampling, as population visiting the GP’s practices is “more ill” and does not fully represent the general population. In this survey, blood pressure therapeutic goals were achieved in more patients from urban than rural settlements and in more patients from the coastal than continental areas. This can be attributed to different patterns of using health care in rural and urban areas (lower accessibility, rare self-measuring control).

Failure to achieve total and LDL-cholesterol target values in asymptomatic participants and in participants with established CVD reported in this survey was worse than previously reported (8-10). This deserves full attention of all the stakeholders in preventive cardiology and public health. It might, at least partly, be explained by the results of a recently published PERCRO study, which showed that although 80% of Croatian physicians (GPs, internists, and cardiologists) believed that they were treating their patients with dyslipidemia well, only 21% of Croatian general population have discussed cholesterol levels with their physician and more than half of them stated that they had never discussed any risk factor with their physician (40,41). This also clearly suggests that a large majority of the participants included in this study would need more intensive cholesterol management to achieve the lipid targets as defined in the guidelines.

In conclusion, the results of this study suggest that Croatia needs a fundamental shift of national health policy priorities toward prevention of CVD, with more active GP’s participation, particularly in continental and rural parts. This would be justified because prevention of CVD has one of the strongest evidence bases of all aspects of medicine (42). Such an approach would reduce the burden of excessive CVD morbidity and mortality in Croatia.

## 

║According to Wolf et al (19).
